# Otitis*Abstract from The Alkaloidal Clinic of Chicago for January, 1899.

**Published:** 1899-05

**Authors:** Hugh Blake Williams

**Affiliations:** Chicago, Ill.


### Otitis.*

BY DR. HUGH BLAKE WILLIAMS, CHICAGO, ILL.

The more I see of chronic suppurative inflammation of the ear, the more convinced do I become that the element of chronicity is due to lack of thoroughness in treatment. The method of procedure mapped out below will not succeed in cases where necrosis has occurred, but in all others it will reduce the duration of treatment from months and weeks to days.

The patient is placed upon the side with the affected ear up. The concha is filled with Marchand's Hydrozone, which is allowed to remain until it becomes heated by contact with the skin, when, by

*Abstract from The Alkaloidal Clinic of Chicago for January, 1899.

tilting the auricle, the fluid is poured gently into the external canal. The froth resulting from the effervescence is removed with absorbent cotton from time to time, and more Hydrozone added. This is kept up until *all* bubbling ceases. The patient will hear the noise even after the effervescence ceases to be visible to the eye.

Closing the external canal by gentle pressure upon the tragus forces the fluid well into the middle ear, and in some instances will carry it through the Eustachian tube into the throat. When effervescence has ceased the canal should be dried with absorbent cotton twisted on a probe and a small amount of pulverized boracic acid insufflated.

The time necessary for the thorough cleansing of a suppurating ear will vary from a few minutes to above an hour, but if done with the proper care it does not have to be repeated in many cases. However, the patient should be seen daily and the Hydrozone used until the desired result is obtained.

Care is necessary in opening the bottle for the first time, as bits of glass may fly. Wrap a cloth about the cork and twist it out by pulling on each side successively.

In children and some adults the Hydrozone causes pain, which can be obviated by previously instilling a few drops of warm solution of cocaine hydrochloride. In this note it has been the intention to treat suppuration of the ear rather as a symptom and from the standpoint of the general practitioner.

